# Antioxidant Properties of Green Plants with Different Vitamin K Contents

**DOI:** 10.3390/molecules29153655

**Published:** 2024-08-01

**Authors:** Iryna Bryshten, Łukasz Paprotny, Małgorzata Olszowy-Tomczyk, Dorota Wianowska

**Affiliations:** 1 Department of Chromatography, Institute of Chemical Sciences, Faculty of Chemistry, Maria Curie-Skłodowska University in Lublin, Pl. Maria Curie-Skłodowska 3, 20-031 Lublin, Poland; 2 Research and Development Centre, ALAB Laboratories, ul. Ceramiczna 1, 20-150 Lublin, Poland

**Keywords:** vitamin K1, phylloquinone, antioxidant activity, DPPH, FRAP, CUPRAC, β-carotene bleaching assay

## Abstract

Vitamin K, as a natural protector of our blood, bones, kidneys, and brain, is essential for human health. It is also considered an effective anti-aging agent with comprehensive biological effects, including antifungal, antibacterial, anti-inflammatory, analgesic, and even antioxidant properties. Of these, the least is known about the antioxidant properties of natural vitamin K. To fill this gap, this study compared the antioxidant properties of extracts obtained from commonly consumed green plants with different vitamin K contents with the activity of vitamin K standard solutions at concentrations corresponding to the vitamin K contents in the extracts. Various measurement methods were used in the research (i.e., DPPH, FRAP, CUPRAC, and the β-carotene bleaching test). Among the tested methods, the β-carotene bleaching test is the most sensitive in the assessment of this unusual compound. In light of the data presented, the antioxidant response of vitamin K alone is dose-dependent. However, in extracts, the activity of this compound is modulated by other constituents present in them. As a result, the activity does not always correlate with vitamin K content. The presented data supplement the knowledge about the antioxidant properties with the contribution resulting from the presence of vitamin K in green plant extracts.

## 1. Introduction

Every living organism is exposed to reactive oxygen species (ROS), including free radicals. These forms are generated, among others, during cellular respiration reactions, destruction of pathogenic microorganisms, or under the influence of physical factors such as ionizing radiation, ultraviolet radiation, and/or ultrasounds [1,2]. Their presence can have a positive or negative impact on the human body, depending on whether there is a balance between their production and sequestration by the body’s antioxidant defense system. When this equilibrium is disturbed because of ROS overproduction and/or reduced efficiency of the defense system, a condition called oxidative stress arises, which is believed to be crucial in the aging process and the development of many diseases, including the cardiovascular system, central nervous system, and malignant conditions [3,4,5,6]. Compounds capable of counteracting and removing the unfavorable effects of ROS are antioxidants which, by participating in the non-enzymatic reaction with free radicals or by donating an electron needed to pair free radicals in enzymatic reduction, lead to the neutralization of these unfavorable forms [6,7].

**Figure 1:**
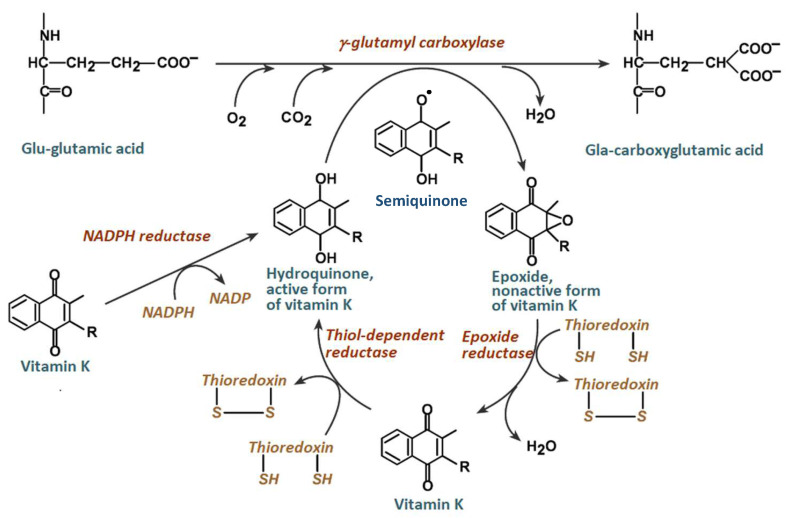
The vitamin K epoxide cycle.

Among the compounds with antioxidant properties, an important position is occupied by those of plant origin, commonly called phytochemicals. In this group, the antioxidant activity is most often associated with phenolic compounds and less frequently with vitamins present in much lower concentrations [8,9,10]. However, while it is widely known about the strong antioxidant properties of vitamins C, E, or A, there are not many studies on the antioxidant effect of vitamin K, especially in plant extracts [11]. In the literature, one can find reports on the antioxidant potential of vitamin K, but these reports are mainly from in vivo studies. These reports almost always focus on the modulation of the redox balance due to the quinone structure of vitamin K. In [12], it was reported that vitamin K inhibits lipid peroxidation in the vitamin K cycle. This cycle is a series of redox transformation processes to which vitamin K is subjected in the tissues of the human body. Its purpose is the carboxylation of glutamic acid (see Figure 1). The antioxidant species of the cycle is vitamin K-hydroquinone, which is converted to the epoxide inactive form of vitamin K. This occurs as a result of the reaction of vitamin K-hydroquinone and a free radical, leading to the formation of the semiquinone radical, formed by the abstraction of γ-glutamyl hydrogen by γ–glutamyl carboxylase. The semiquinone reacts with superoxide anion or oxygen to form a reactive intermediate that breaks the γ-glutamyl hydrogen bond simultaneously with the ring epoxidation, resulting in the formation of an inactive form of vitamin K epoxide. This form is then reduced to quinone by vitamin K epoxide reductase. The quinone is, in turn, converted into hydroquinone by reductase in the presence of NADPH and can again participate in another cycle of redox transformation [8,13]. In [14], it was shown that the fully reduced form of vitamin K protects cells not only from harmful lipid peroxidation but also from ferroptosis, a non-apoptotic form of cell death characterized by iron-dependent lipid peroxidation, which plays a key role in organ damage, degenerative disease and susceptibility to treatment-resistant cancers. Nevertheless, vitamin K has many health-promoting properties. The most important of them is the prevention of osteoporosis and cardiovascular diseases [10]. These diseases usually occur in old age; hence, relatively recently, vitamin K has been recognized as an effective anti-aging factor. In this context, it is not surprising that there is so much interest in its biological activity and extraction as well as analysis of vitamin K in plants, food products, dietary supplements, and bio-fluids [10,15,16].

**Figure 2:**
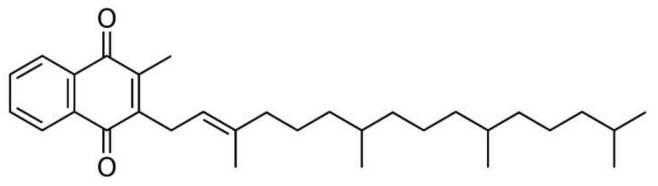
Chemical structure of vitamin K1 (phylloquinone).

Vitamin K is a family of compounds with a similar structure, the core of which is a naphthoquinone ring containing an isoprenoid side chain (R in Figure 1) in the three-position of various lengths and degrees of saturation. Generally, there are three fat-soluble vitamers of vitamin K (i.e., K1, K2, and K3). Of all the forms, the most important role in the human diet is played by vitamer K1, commonly called phylloquinone or phytomenadione, constituting over 80% of vitamin K in the diet [6,8,10]. The molecule of this compound is characterized by the presence of 4 isoprenoid residues in the side chain, one of which is unsaturated (Figure 2). This compound is the final product of the shikimate pathway in the photosynthesis process; hence, it occurs at various concentration levels in many plants. Relatively large amounts of it are found in green plants, which results from the correlation of the amount of this vitamin with the amount of chlorophyll. The discrepancy between the relatively common occurrence of vitamin K1 in nature and the paucity of information on its antioxidant properties and participation in the natural antioxidant protection of our body resulted in research, the results of which are presented and discussed in this paper.

The question posed in this study is to what extent (if at all) vitamin K1 is responsible for the antioxidant properties of green plant extracts. To answer this question, commonly consumed green plants were used. The research was carried out in two blocks. In the first one, in order to select the most sensitive method for assessing the antioxidant activity of extracts, their biological activity was compared using various spectrophotometric methods. In the second, using a selected method, the antioxidant properties of extracts were compared with the properties of solutions of the vitamin K1 standard itself. The research used extracts obtained with one of the modern assisted extraction techniques, namely the ultrasound-assisted solvent extraction (UASE) technique. Liquid chromatography coupled with mass spectrometry (LC-MS/MS) was used to determine the content of vitamin K1 in the extracts.

## 2. Results and Discussion

### 2.1. Extraction and Determination of Vitamin K1 Content in Tested Plant Materials

Extraction of vitamin K1 from plant material was carried out in the optimized ultrasound-assisted extraction conditions (UASE); that is, extraction solvent—*n*-hexane/ethyl acetate mixture (4:1, *v*/*v*), extraction temperature—50 °C, ultrasound frequency—37 kHz, generator power—100%, extraction time—20 min, and ratio of plant material to the volume of the extraction mixture—0.1/15 g/mL. The description of validation experiments is in Section 3.2. Contrary to the data presented in [15], freeze-dried material was subjected to extraction. The LC-MS/MS analysis of the obtained extracts was carried out in accordance with the previously presented procedure [16] with small adaptations described in Section 3.3. The obtained results are presented in Table 1. The data were expressed as the content of vitamin K1 in μg per g of material (column 3) and as the concentration of vitamin K1 in the extract (in μg per mL, column 4). Numbers 1–13 (column 1) refer to specific vegetables whose names are in column 2 of the table. As shown by the data presented below, plant material chosen for research is characterized by a different content of vitamin K1. Spinach contains the most K1 vitamer—111.70 [μg/g]. Avocado has the lowest content of this vitamin—0.67 [μg/g]. Other vegetables reach values from 1.56 [μg/g] for broad beans to 76.39 [μg/g] for arugula.

### 2.2. Determination of Antioxidant Properties of Plant Extract by Using Different Spectrophotometric Methods

To select the best method for assessing the antioxidant properties of extracts from green plants with different contents of vitamin K1 and vitamin K1 itself, the antioxidant properties of extracts were assessed based on various research methods measuring different types of antioxidant activity, including the ability to neutralize radicals (DPPH method), the ability to reduce metal ions (FRAP and CUPRAC methods), and the ability to protect against oxidation of other molecules (β-carotene bleaching method). The obtained results are presented in the following subsections.

#### 2.2.1. Assessment of Extract Activity Using the DPPH Method

**Figure 3:**
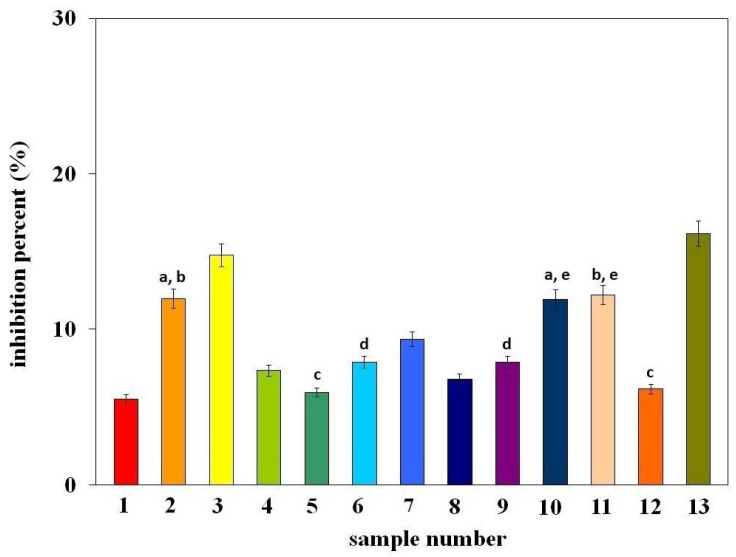
Antioxidant activity, expressed as % inhibition of the DPPH radical, obtained for the examined extracts with different content of vitamin K1. The numbers from 1–13 correspond to the sample numbers from Table 1. The letters in the figure indicate data for which the difference in %I is not statistically significant (Fcal < Ftab, *p* > 0.05).

The DPPH method is one of the most frequently used; therefore, research on the antioxidant properties of green plant extracts began with this method. The obtained results are shown in Figure 3 as the % inhibition (%I) of the DPPH radical, determined after 60 min of radical neutralization by the tested extracts. The numbers on the X-axis from 1–13 correspond to the sample numbers from Table 1. The letters in the figure indicate data for which the difference in %I is not statistically significant (Fcal < Ftab, *p* > 0.05).

As can be seen from the presented data, the antioxidant properties of the tested extracts are diverse. The highest antioxidant activity is demonstrated by extracts from spinach (system number 13), broccoli (system number 3), iceberg lettuce (system number 11), broad beans (system number 2), and arugula (system number 10). The % inhibition for the remaining samples is below 10%. It should be noted here that, in general, all the results obtained are low. This may indicate that such a popular method of assessing antioxidant properties is not adequate to assess the activity of the tested extracts.

According to literature data [17], the DPPH radical is neutralized either as a result of the addition of an electron and hydrogen from the antioxidant (as is the case with phenolic antioxidants) or as a result of attachment to a double bond in the structure of the antioxidant molecule, forming an adduct with it. The vitamin K1 molecule (Figure 2) does not contain any hydroxyl group in its structure, only a double bond, which indicates that its antioxidant properties in the DPPH method will result from the ability to coordinate a radical to the double bond [18]. However, in light of the data presented, this hypothesis is unlikely due to the very weak antioxidant activity of the extracts. It is also probable that the method of neutralizing the DPPH radical differentiates the antioxidant activity of the analyte. Nevertheless, when comparing the antioxidant properties of extracts with the content of vitamin K1 (Table 1), it can be seen that there is no correlation between them. It seems that the weak antioxidant properties obtained for the examined extracts may result from the use of the *n*-hexane/ethyl acetate mixture as an extractant and its poorer ability to isolate compounds with higher antioxidant potential (e.g., polyphenol compounds). This hypothesis is confirmed by the results obtained by Yasin et al. [19], who determined the antioxidant properties of alcohol extracts from food sources rich in vitamin K (e.g., spinach), indicating that they result mainly from the content of phenolic compounds in the tested extracts, and not from the vitamin K content.

#### 2.2.2. Evaluation of the Activity of Extracts Using the FRAP and CUPRAC Methods

**Figure 4:**
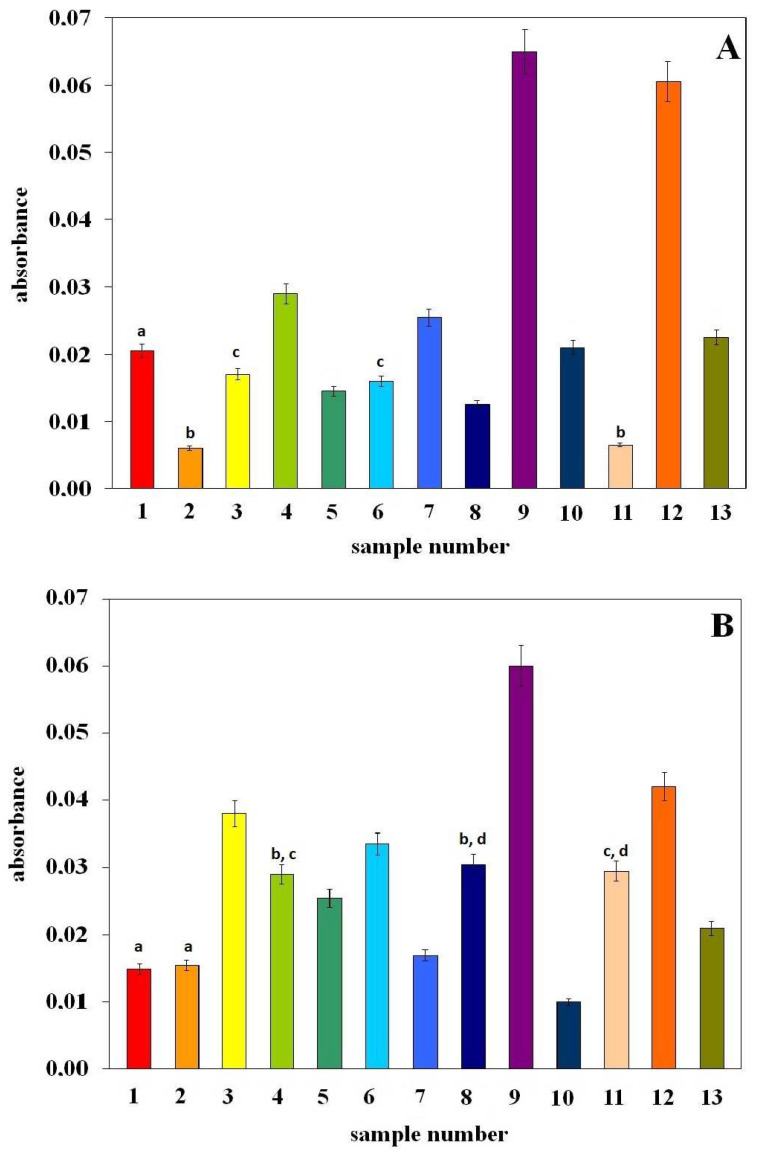
Antioxidant activity expressed as absorbance of values of the colored complex obtained in FRAP (**A**) and CUPRAC (**B**) assays. The numbers from 1–13 correspond to the sample numbers from Table 1. The letters in the figure indicate data for which the difference in %I is not statistically significant (Fcal < Ftab, *p* > 0.05).

Figure 4 shows the antioxidant properties of examined extracts assessed using the FRAP (Ferric Reducing Antioxidant Parameter) and CUPRAC (Cupric Ion Reducing Antioxidant Capacity) methods. The essence of their measurement is similar and consists of the ability to reduce metal ions in the complexes in which they occur [20,21]. Hence, in the figure mentioned, the antioxidant properties are expressed as absorbance values of the colored complex formed during the reduction reaction.

As shown by the data presented in Figure 4, both methods show very low absorbance values, which proves the low metal reduction capabilities of the extracts. Therefore, the tested extracts show weak electron-donating activity—low absorbance values indicate a small number of complexes formed. It should also be noted that in both methods, samples no. 9 (parsley extract) and no. 12 (chives extract) have the greatest reduction capacity. The remaining extracts have lesser properties, depending on the method. The FRAP and CUPRAC methods, despite the same measurement idea (metal reduction), are not identical because the measurement in both methods takes place in different measurement systems (different pH values; FRAP method—pH = 3.6 and CUPRAC—pH = 7) [18]. This fact may explain the observed differences in the antioxidant properties of the tested extracts. It should be added that, similarly to the DPPH method, in the case of FRAP and CUPRAC there is no correlation between activity and vitamin K content.

According to the literature [22], the ability of compounds to reduce heavy metal ions leads to the generation of highly reactive and cytotoxic hydroxyl and hydroperoxyl radicals as a result of the Fenton reaction. It has been shown that menadione, through the production of hydroxyl radicals and DNA strand breakage, causes oxidative stress in cancer cells. A similar pro-oxidant effect has been observed for vitamins C and E [11]. Nevertheless, the main function of vitamin C is its antioxidant effect. It is similar in the case of vitamin E, which, on the one hand, protects cell membranes from lipid peroxidation and, on the other hand, can reduce Fe or Cu as a pro-oxidant. The ability of both vitamins to act as a pro- or antioxidant depends on the amount available for ROS removal. In other words, in general, if the amount of a given compound is adequate to cause a decrease in the concentration of ROS, the antioxidant effect prevails. If the amount of the compound is inadequate, an environment favorable to the promotion of apoptosis is created [22]. Our results show a low ability of extracts to reduce iron and copper ions, which may indicate a low susceptibility to the Fenton reaction.

Summarizing the results obtained for extracts using the DPPH, FRAP, and CUPRAC methods, it can be concluded that the examined extracts have weak antioxidant properties. The obtained results also indicate the lack of a positive correlation between the vitamin K1 content in the examined extracts and their antioxidant properties. Taking the above into account, it was decided to check what properties the extracts show in the β-carotene bleaching assay (i.e., in a system in which the model antioxidant used (β-carotene), like vitamin K, has double bonds in its structure).

#### 2.2.3. Assessment of Antioxidant Activity by β-Carotene Bleaching Method

**Figure 5:**
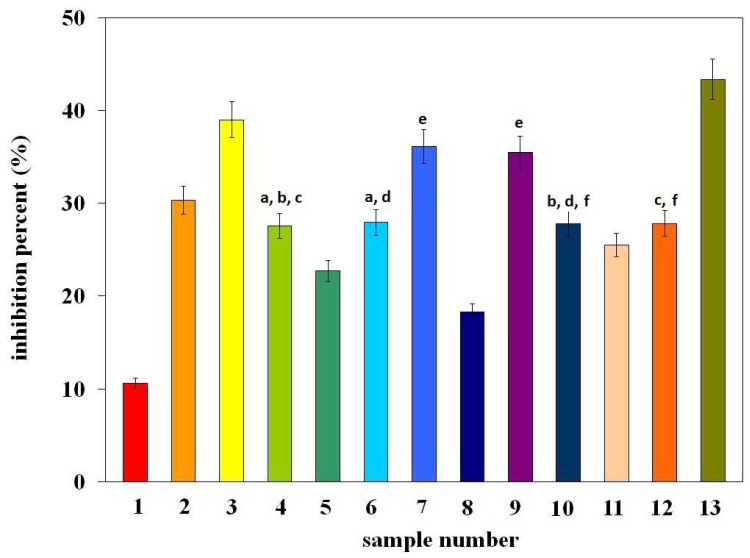
% inhibition of the sample (containing β-carotene and the examined extract) relative to the control sample (containing only β-carotene itself). Explanation of the meaning of the numbers—see Table 1. The letters in the figure indicate data for which the difference in %I is not statistically significant (Fcal < Ftab, *p* > 0.05).

Figure 5 shows the antioxidant properties expressed as % inhibition of the sample (containing β-carotene and the examined extract) relative to the control sample (containing only β-carotene itself). In other words, the values on the Y-axis represent the % inhibition of β-carotene oxidation in the sample with the examined extract in relation to the control sample without the examined extract. The numbers on the X-axis from 1–13, as before, correspond to the sample numbers from Table 1.

According to the presented data, the highest antioxidant activity is demonstrated by the following samples: no. 13—spinach extract (43.35%), no. 3—broccoli extract (39, 00%), no. 7—cucumber extract (36.11%), no. 9—parsley extract (35.45%), and no. 2—broad bean extract (30.34%). The remaining samples show values below 30%. It should be emphasized that the results obtained compared to the results of other methods (e.g., % inhibition obtained by the DPPH method) are much higher, which indicates that this method is more suitable for assessing the antioxidant properties of the examined extracts. This may be due to the structural similarity of vitamin K1 (contained in each extract) and β-carotene. According to the literature [23], β-carotene, due to its double bonds in its structure, has the ability to form radical adducts with LOO radicals resulting from the oxidation of linoleic acid. It seems that vitamin K1, due to its structural similarity to β-carotene, behaves similarly. Therefore, further research (aimed at comparing the antioxidant properties of the extract with the antioxidant properties of a vitamin K1 solution, which contains the same amount of vitamin K as in a given extract) was decided to be performed using the β-carotene bleaching method.

### 2.3. Comparison of the Antioxidant Properties of Extracts with the Antioxidant Properties of Vitamin K1 Solutions Using the β-Carotene Bleaching Test

**Figure 6:**
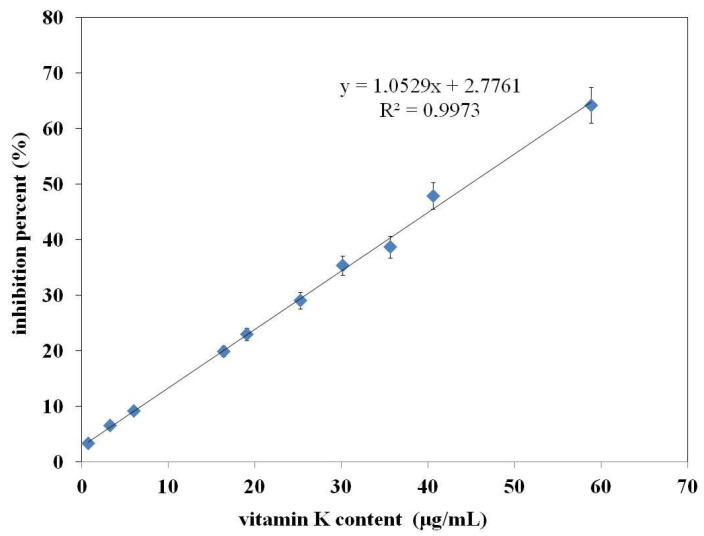
% inhibition of the sample (containing β-carotene and the examined vitamin K solution) relative to the control sample (containing only β-carotene itself) for vitamin K solutions at different concentrations.

In order to verify the hypothesis that the structural similarity of vitamin K1 and the model antioxidant (β-carotene) may be responsible for the better antioxidant properties of extracts tested using the β-carotene bleaching method, in the next stage of the research, it was decided to check the antioxidant properties of pure vitamin K1. For this purpose, the antioxidant properties of vitamin K1 solutions with different concentrations were tested. The results of these experiments are presented in Figure 6.

**Figure 7:**
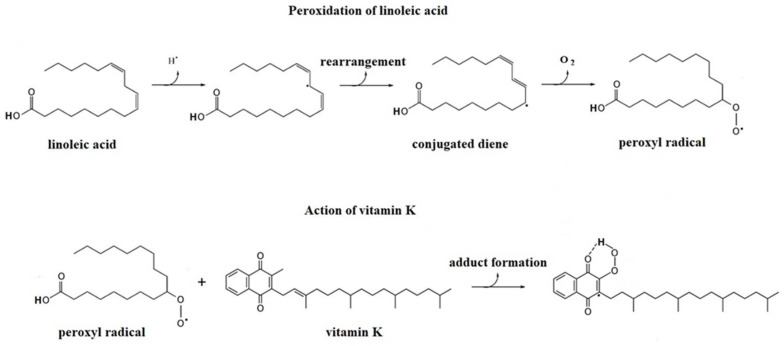
Proposed radical addition reaction pathway for the antioxidant activity of vitamin K1.

Figure 6 shows the dependence of the % inhibition on the vitamin K content. The range of vitamin K solutions used covers the range of vitamin K concentrations in the extracts tested (see Table 1). As you can see, the dependency is linear. This means that as the concentration increases, greater antioxidant activity of vitamin K is observed. In other words, these experiments have proven that vitamin K1 has antioxidant properties. The proposed radical addition reaction pathway for the antioxidant activity of vitamin K1, in a way other than modulation of the redox balance due to the quinone structure of vitamin K, is presented in Figure 7. As shown, lipids such as linoleic acid form a peroxyl radical in the presence of ROS and/or O_2_. This radical reacts with vitamin K1 to form a stable vitamin radical. As a result, inhibition of β-carotene oxidation is observed.

Taking the above into account, in the next course of research, it was decided to check whether the antioxidant properties of the examined extracts resulted from their vitamin K content. For this purpose, the experiments were carried out to estimate the % inhibition for vitamin K solutions in which the vitamin K content was the same as in the extracts tested (see Table 1). For clarity, as shown in the table, spinach extract contains 111.70 μg of vitamin K per g of vegetables, which corresponds to a vitamin K concentration in the extract of c = 11.78 μg/mL. By comparing the % inhibition obtained for the spinach extract and the % inhibition for the vitamin K solution with a concentration of c = 11.78 μg/mL, it can be determined whether the antioxidant properties of the extract depend on the vitamin K content.

**Figure 8:**
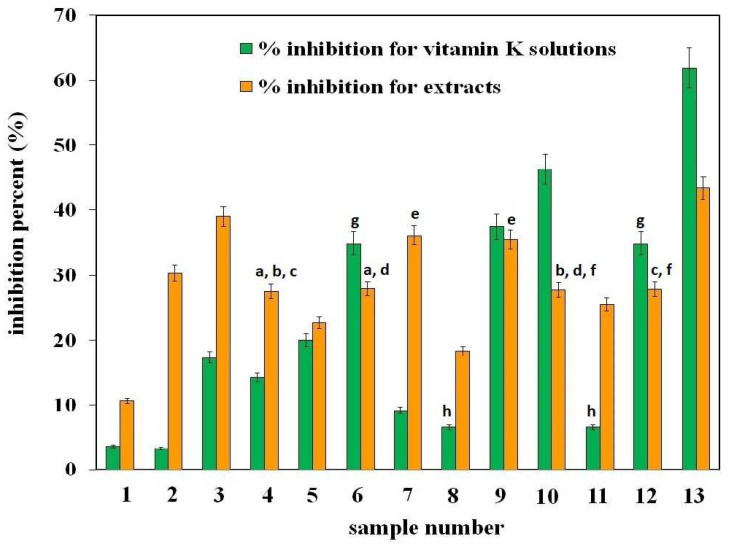
Antioxidant properties expressed as the inhibition percentage obtained for vitamin K solutions (green bars) and for the examined plant extracts (orange bars). The letters in the figure indicate data for which the difference in %I is not statistically significant (Fcal < Ftab, *p* > 0.05).

A comparison of the inhibition percentage obtained for individual extracts (orange bars) and for vitamin K solutions (green bars) is shown in Figure 8. The results are given in pairs. In each of them, the orange bar represents the % inhibition of the extract, and the green bar corresponds to the % inhibition of a vitamin K solution with the same concentration as in the extract.

As can be seen from the presented data, the heights of the bars in individual pairs are not the same. This proves that not only vitamin K is responsible for the antioxidant properties of the extract but also its other ingredients, which may increase its antioxidant properties compared to the activity of vitamin K alone (pairs no. 1–5, 7, 8, and 11) or decrease them (i.e., they suppress the effect of vitamin K itself, what is observed for pairs 6, 9, 10, 12, and 13). The obtained results, independently from the method of assessing antioxidant properties, confirm the lack of correlation between the content of vitamin K1 and the antioxidant properties of extracts. It is true that tests carried out on a simple system (i.e., on a vitamin K1 standard solution) showed that the more vitamin K, the better the antioxidant properties of the solution. However, in the case of real samples of extracts that are very complex mixtures, such correlation was not observed, as already mentioned. Other components of extracts may affect the activity of vitamin K1 in an additive, antagonistic, and/or synergistic manner. As a result, the resultant effect will be different from that observed in a simple system [24]. This is a phenomenon known from the literature and explains why it is difficult to clearly explain and predict what the final effect of the mixture will be. This highlights the complexity of understanding the beneficial effects of foods beyond their individual antioxidant components.

## 3. Materials and Methods

### 3.1. Plant Materials and Chemicals

The following vegetables and fruits were used in the experiments: iceberg lettuce (*Lactuca sativa var. capitata*), cucumber (*Cucumis sativus*), spinach (*Spinacia oleracea*), broccoli (*Brassica oleracea var. italica*), bean (*Vicia faba*), bell pepper (*Capsicum annuum*), arugula (*Eruca vesicaria ssp. sativa*), kale (*Brassica oleracea*), brussels sprouts (*Brassica oleracea var. gemmifera*), chives (*Allium schoenoprasum*), parsley (*Petroselinum crispum*), dill (*Anethum graveolens* L.), and avocado (*Persea americana* Mill.). All of them were purchased from local stores (Lublin, Poland). Samples were freeze-dried using Labconco Centrivap Cold Trap. For this purpose, plants were pre-frozen in a freezer and then subjected to a lyophilization process, during which moisture was checked every 50 min. The process was continued until the change in mass loss between consecutive measurements was less than 0.1 g. Then, the dried samples were ground with a Braun cutting mill to a particle size of 0.2–0.4 mm. Thoroughly weighted portions of the samples were used for extractions.

CuCl_2_, FeCl_3_·6H_2_O, HCl, CH_3_COONH_4_, ethanol, ethyl acetate, *n*-hexane, ethyl acetate, methanol, CH_3_COONa, and CH_3_COOH were purchased from the Polish Chemical Plant POCh (Gliwice, Poland). 2,2′-Diphenylpicrylhydrazyl (DPPH), formic acid (LC-MS grade), linoleic acid, Tween 20, neocuproine (2,9-dimethyl-1,10-phenanthroline, Nc), β-carotene, methanol (HPLC grade), 2,4,6-tri(2-pyridyl)-s-triazine (TPTZ), vitamin K₁ (phytomenadione) and deuterium-labeled K2MK-7-D7 were purchased from Sigma Aldrich (Poznań, Poland). Water was purified using the Milli-Q system (Millipore Sigma, Bedford, MA, USA).

### 3.2. Ultrasound-Assisted Solvent Extraction

UASE was performed in an Elmasonic P ultrasonic bath (Elma, Singen, Germany) under controlled and optimized extraction conditions: extraction time—20 min, frequency—37 kHz, temperature—50 °C, power—100% using iceberg lettuce and *n*-hexane/ethyl acetate extraction mixture (4:1, *v*/*v*). During the experiments, the influence of the type of solvent (methanol, acetone, n-hexane/ethyl acetate (4/1, *v*/*v*), n-hexane), temperature (ambient or 50 °C) extraction time (5 min, 10 min, 15 min, 20 min), ultrasound frequency—37 or 80 kHz, and the power of the ultrasonic generator (50 or 100%) were checked on the isolation efficiency of vitamin K1. For the extraction, portions of freeze-dried plant samples (0.1 g) were placed in tightly screwed centrifuge tubes with 15 mL of the extraction mixture. In order to separate the obtained extract from the plant material, centrifugation (10 min, 2700 rpm) was used. Then, the samples were poured into 50 mL volumetric flasks, which were filled up to the volume with the extraction mixture. Prior to analysis, 1 mL of the extract was evaporated to dryness under a stream of nitrogen and then reconstituted in the same volume of methanol with 0.1% formic acid.

### 3.3. Chromatographic Analysis of Vitamin K1

Analyses were performed according to the previously described procedure [16] with adaptations. As before, the measurements were performed on a Shimadzu NEXERA X2 LC instrument (Shimadzu, Kyoto, Japan) consisting of a double binary pump, a system controller, an automatic solvent degasser, and an autosampler. The autosampler temperature was maintained at 20 °C. Separations were performed on a Kinetex C18 column (50 × 2.1 mm, 2.6 µm, Phenomenex, Inc., Torrance, CA, USA) using a modified gradient program with a mobile phase flow at 0.6 mL/min. The mobile phase A was a 0.1% solution of formic acid in water; the mobile phase B was 0.1% formic acid in MeOH. The gradient program started with an increase from 90% B to 100% B in 4 min, then 100% B was maintained for another 4 min, and finally, the column was equilibrated with the initial composition of the mobile phase, i.e., 90% B for 10 min. The total run time was 18 min, with the first 8 min to be monitored. The column temperature was maintained at 40 °C and controlled by a thermostat. The volume of the injected sample was reduced to 2 µL. Detection was performed on a triple quadrupole mass spectrometer LCMS-8050 (Shimadzu, Kyoto, Japan) equipped with an atmospheric pressure chemical ionization (APCI) source operating in the positive ion mode under the following conditions: APCI temperature 350 °C, desolvation line temperature 200 °C, heat block temperature 200 °C, gas flow from the nebulizer 3 [L/min] and drying gas flow 5 [L/min].

Identification and quantification were based on MS/MS multiple reaction monitoring (MRM) mode after examining the fragmentation spectrum of the analyte in accordance with the identity confirmation criteria taken from Commission Decision 2002/657/EC. According to them, MRM measurements for vitamin K1 were carried out for two transitions: *m*/*z+* 451.25 → 105.05 (qualifier transition, S2) and 451.25 → 187.10 (quantifier transition, S1) and one transition *m*/*z* + 656.0 → 194.1 for K2MK-7-D7 using the collision energy (CE) values at 45 eV, 30 eV, and 35 eV, respectively. Due to the high price of deuterium-labeled standards, quantitative analysis, as before, was performed using the K2MK-7-D7 standard as an internal standard.

### 3.4. Antioxidant Activity Measurements

The antioxidant properties of the obtained extracts and vitamin K1 standard solutions were assessed spectrophotometrically using the four methods presented below. All measurements were performed using a UV Probe-2550 Spectrophotometer (Shimadzu, Kyoto, Japan) and an optical glass cuvette (1cm ×1 cm × 3.5 cm).

#### 3.4.1. DPPH Method

The estimation of the remaining DPPH radical concentration in DPPH solution after reaction with the examined extract was performed using a slightly modified procedure by Brand-Williams et al. [25]. Briefly, 100 µL of tested plant extracts dissolved in a mixture containing *n*-hexane: ethyl acetate (in the ratio 4:1, *v*/*v*) was mixed with DPPH solution (2900 µL) in a 4 mL test tube. The mixture was stirred vigorously for 30 s, poured into the optical cuvettes, and immediately placed in a spectrophotometer. The changes of absorbance at 516 nm were monitored for 60 min at room temperature. Subsequent readings were taken at regular time intervals (1 min). The inhibition percent (I %) was calculated from the following equation:

(1) $$I\left(\%\right)=\left(1-\frac{A_{60}}{A_{0}}\right)\times 100\%$$

where: A_60_ and A_0_ are the absorbance values of DPPH^•^ at 0 and 60 min of the radical neutralization reaction, respectively.

A mixture containing 2900 µL of methanol and 100 µL of extraction solvent (*n*-hexane: ethyl acetate (4:1, *v*/*v*)) was used to zero the spectrophotometer.

#### 3.4.2. FRAP Method

A FRAP assay was carried out using the method of Benzie and Strain [26]. The FRAP test solution was prepared using FeCl_3_·6H_2_O in distilled water (the final concentration of Fe(III) in the solution was 20 mM), 2,4,6-tri(2-pyridyl)-s-triazine (TPTZ) in 40 mM HCl (final concentration of TPTZ was 10 mM), and 0.3 M CH_3_COOH/CH_3_COONa buffer solution at pH = 3.6. The FRAP reagent was prepared daily as follows: acetic acid buffer, TPTZ solution, and FRAP test solution were mixed in this order at the volume ratio of 10:1:1.

A 2900 µL aliquot of FRAP reagent was mixed in a 4 mL test tube with 100 µL of tested plant extracts dissolved in a mixture containing *n*-hexane: ethyl acetate (in the ratio 4:1, *v*/*v*). The mixture was vigorously shaken for 30 s and left in the dark for 60 min at 37 °C, which was necessary to ensure the best electron transfer from the sample to the iron. It was subsequently poured into an optical glass cuvette and immediately placed in a spectrophotometer to measure the increase of absorbance at 593 nm.

A mixture containing 2900 µL of FRAP reagent and 100 µL of extraction solvent (*n*-hexane: ethyl acetate (4:1, *v*/*v*)) was used to zero the spectrophotometer. The antioxidant properties were expressed as the absorbance values of the colored complex formed during the reaction after the reduction of Fe (III) by antioxidants.

#### 3.4.3. CUPRAC Method

The CUPRAC method is based on the reduction of the cupric neocuproine complex (Cu(II)-Nc) to the cuprous form (Cu(I)-Nc) by examined antioxidants [27]. In this method, the applied CUPRAC test solution is usually prepared using CuCl_2_ (final concentration of Cu(II) in the solution was 10 mM), neocuproine in absolute ethanol (final concentration 7.5 mM), and 1.0 M CH_3_COOH/CH_3_COONH_4_ buffer solution at pH = 7.0. The reagents are measured as follows: 500 µL of Cu(II) solution+ 500 µL of Nc solution + 500 µL of buffer + 100 µL of tested plant extracts dissolved in a mixture containing hexane: ethyl acetate (in the ratio 4:1, *v*/*v*). + 450 µL of water. The obtained mixture was vigorously shaken for 30 s and left in the dark for 60 min. Then, it was poured into an optical glass cuvette and immediately placed in a spectrophotometer to measure the increase in absorbance at 450 nm at room temperature.

A mixture containing all reagents and 100 µL of extraction solvent (*n*-hexane: ethyl acetate (4:1, *v*/*v*)) was used to zero the spectrophotometer. The antioxidant properties were expressed as the absorbance values of the colored complex formed during the reaction after the reduction of Cu (II) by antioxidants.

#### 3.4.4. β-Carotene Bleaching Assay

A slightly modified Dapkevicius method [28] was applied for the estimation of the reacted β-carotene concentration in the examined systems. The preparation of the stock solution of β-carotene/linoleic acid emulsion in water was as follows: the mixture composed of 25 μL of linoleic acid, 185 μL of Tween 20 (200 mg), and 5 mL of β-carotene solution (containing 0.5 mg of β-carotene in 1 mL of chloroform) was vacuum evaporated to remove chloroform. The residue was dispersed in 100 mL of distilled water saturated with oxygen for 30 min (the flow rate of oxygen during the saturation process was equal to 100 mL/min). The mixture was vigorously shaken, and a 2900 μL volume was placed in a cuvette along with 100 μL of tested plant extracts dissolved in a mixture containing *n*-hexane: ethyl acetate (in the ratio 4:1, *v*/*v*). The cuvette was stoppered tightly and mixed, then (with emulsion and antioxidant) was placed in a water bath. The measurements were carried out at a temperature of 45 °C to induce spontaneous oxidation of linoleic acid, generating radicals that discolor the aqueous emulsion of linoleic acid and β-carotene [29]. The changes in β-carotene absorbance were monitored at 470 nm. As a blank, the emulsion without β-carotene was applied in measurements. The first measurement (after mixing of components) was assumed to show a 0 reading. The subsequent measurement readings were taken at constant intervals (10 min) until the orange color of the control sample disappeared (about 180 min). The control sample was prepared in the same way as the reacting mixture.

To zero the spectrophotometer, the mixture containing 100 µL of extraction solvent (n-hexane: ethyl acetate, 4:1, *v*/*v*) and 2900 µL of the emulsion without β-carotene was used. The antioxidant properties (I%) of the sample (containing β-carotene and the examined extract or vitamin K1 solution) were expressed relative to the control sample (containing only β-carotene itself). The following equation was used:

(2) $$I\left(\%\right)=100\times \frac{{\mathrm{DR}}_{C}-{\mathrm{DR}}_{S}}{{\mathrm{DR}}_{c}}$$

where: I(%)—inhibition percent, DR_C_—degradation rate of β-carotene in the control sample = {[ln (a/b)]/t}, DR_S_—degradation rate of β-carotene in the sample with antioxidant = {[ln (a/b)]/t}, a = absorbance at 0 min, b = absorbance at defined time (for example at 10, 20 …. to 180 min), and t = time.

### 3.5. Statistical Analysis

All results are presented as the mean value of five independent measurements unless otherwise stated (n = 5) ± standard deviation (SD). The one-way analysis of variance (ANOVA) and Fisher coefficient (*F*) value were used to assess the activity. Antioxidant activity differences in the studied groups were considered significant for *p* ≤ 0.05 significance level and *Fcrit < Fcal*. The statistical analysis was performed using Excel (Microsoft Excel 2010).

## 4. Conclusions

The paper presents and discusses the antioxidant properties of green plant extracts with different vitamin K1 contents. Based on the low values of the inhibition percentage in the DPPH method and the absorbance of color complexes in the FRAP and CUPRAC methods, it was shown that the antioxidant properties of the tested extracts determined by these three methods are low. Due to the better antioxidant properties of extracts demonstrated by the β-carotene method, it was found that this method is more adequate for assessing the antioxidant properties of green plant extracts. Using the β-carotene method, it was also proven that vitamin K1 itself has dose-dependent antioxidant properties, and with an increase in its concentration in the solution, an increase in antioxidant activity is observed. Comparison of the antioxidant properties of extracts with different contents of vitamin K1 and standard solutions of vitamin K1 with a concentration identical to the concentration of this analyte in the extract allows us to conclude that in the case of a mixture, other ingredients modulate the action of vitamin K1 through synergistic, antagonistic and/or additive effects. This highlights the complexity of understanding the beneficial effects of foods beyond their individual antioxidant components. In times of great interest in the health-promoting properties of vitamin K, the results obtained will expand knowledge about them. In addition, they will contribute to increasing public awareness of natural mixtures of antioxidants with vitamin K1.

## Figures and Tables

**Table 1:** The content of vitamin K1 in the tested extracts obtained from various freeze-dried green fruits and vegetables. Values are expressed in μg per g of material or μg per mL of extract. All results are the average of three independent measurements.

Sample No	Vegetable Name	The Content of Vitamin K1	
		μg/g	μg/mL
1	Avocado	0.67 ± 0.07	0.07 ± 0.01
2	Broad bean	1.56 ± 0.05	0.16 ± 0.02
3	Broccoli	36.54 ± 1.10	3.82 ± 0.11
4	Brussel sprouts	30.25 ± 0.91	3.28 ± 0.10
5	Kale	51.71 ± 1.55	5.05 ± 0.16
6	Dill	55.86 ± 1.68	6.06 ± 0.18
7	Cucumber	11.26 ± 0.34	1.21 ± 0.04
8	Pepper	5.93 ± 0.18	0.62 ± 0.03
9	Parsley	69.70 ± 2.09	7.14 ± 0.21
10	Arugula	76.39 ± 2.29	8.13 ± 0.24
11	Iceberg salad	14.82 ± 0.45	0.66 ± 0.02
12	Chives	56.65 ± 1.70	6.03 ± 0.18
13	Spinach	111.70 ± 3.35	11.78 ± 0.35

## Data Availability

The original contributions presented in the study are included in the article, further inquiries can be directed to the corresponding authors.
